# In Silico Analysis of the Gene Expression Patterns between Aldosterone-Producing Adenoma and Nonfunctional Adrenocortical Adenoma

**DOI:** 10.1155/2021/9553637

**Published:** 2021-10-04

**Authors:** Yongfa Dai, Jing Li, Hong Wen, Jie Liu, Jianling Li

**Affiliations:** ^1^Department of Cardiovascular Medicine, The First Nanning People's Hospital, Nanning, Guangxi 530016, China; ^2^Department of Nuclear Medicine, The First Affiliated Hospital of Guangxi Medical University, Nanning, Guangxi 530031, China; ^3^Department of Cardiovascular Medicine, The First Affiliated Hospital of Guangxi Medical University, Nanning, Guangxi 530021, China; ^4^Postdoctoral Mobile Station of Guangxi Medical University, Nanning, Guangxi 530021, China

## Abstract

Primary aldosteronism is the most common form of secondary hypertension, and aldosteronoma makes up a significant proportion of primary aldosteronism cases. Aldosteronoma is also called aldosterone-producing adenoma (APA). Although there have been many studies about APA, the pathogenesis of this disease is not yet fully understood. In this study, we aimed to find out the difference of gene expression patterns between APA and nonfunctional adrenocortical adenoma (NFAA) using a weighted gene coexpression network (WGCNA) and differentially expressed gene (DEG) analysis; only the genes that meet the corresponding standards of both methods were defined as real hub genes and then used for further analysis. Twenty-nine real hub genes were found out, most of which were enriched in the phospholipid metabolic process. *WISP2*, *S100A10*, *SSTR5-AS1*, *SLC29A1*, *APOC1*, and *SLITRK4* are six real hub genes with the same gene expression pattern between the combined and validation datasets, three of which indirectly or directly participate in lipid metabolism including *WISP2*, *S100A10*, and *APOC1*. According to the gene expression pattern of DEGs, we speculated five candidate drugs with potential therapeutic value for APA, one of which is cycloheximide, an inhibitor for phospholipid biosynthesis. All the evidence suggests that phospholipid metabolism may be an important pathophysiological mechanism for APA. Our study provides a new perspective regarding the pathophysiological mechanism of APA and offers some small molecules that may possibly be effective drugs against APA.

## 1. Introduction

**Figure 1:**
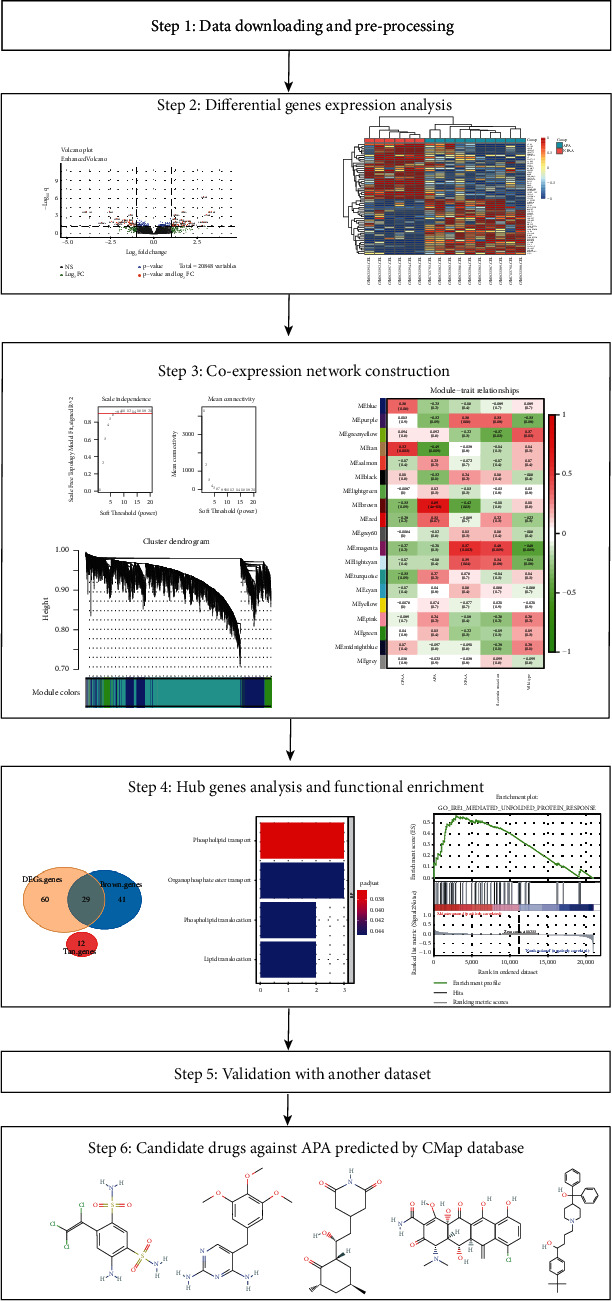
Workflow of the study.

Primary aldosteronism (PA) is now widely recognized as the common causative agent of hypertension cases, especially for treatment-resistant hypertension [1]. Compared to essential hypertension, PA increases the risk of cardiovascular diseases such as stroke, coronary artery disease, atrial fibrillation, and heart failure [2]. The currently accepted prevalence rate of PA is 5–10% among hypertension patients, but the actual prevalence rate might be 3–5 times higher than the currently supposed rate [3]. PA is mainly caused by aldosterone-producing adenoma (APA) [4], an adrenal tumor that excessively secretes aldosterone, which leads to hypertension and hyperkalemia. Certain adrenocortical adenomas, called nonfunctional adrenocortical adenoma (NFAA), do not secrete hormones but simply manifest as a small lump on the upper pole of the kidney. A nationwide multicenter study in Japan revealed that 50.8% of total adrenal incidentalomas were NFAA, while only 5.1% were APA [5]. This study aimed to find out the differences of the gene expression pattern between these kinds of states which may let us further understand the pathogenesis of APA. Previous studies have identified somatic mutations in the potassium channel gene *KCNJ5* and the calcium channel gene *CACNA1D*, which contribute to aldosterone production and cell proliferation in adrenal glomerulosa [6, 7]. Our study mostly focuses on the gene expression level of APA and NFAA. It has been acknowledged that unilateral forms of PA can be treated by adrenalectomy, while bilateral disease only can be treated by medical therapy based on mineralocorticoid receptor antagonists [8]. Since adrenalectomy is not suitable for bilateral APA and mineralocorticoid receptor antagonists are associated with various sex-steroid-related adverse effects, mainly breast tenderness, menstrual abnormalities, decreased libido, and impotence, especially when used at high doses, development of new drugs for the treatment of APA is necessary [9]. According to one of the latest studies, mineralocorticoid receptor antagonists might decrease the rates of positive screening for primary aldosteronism [10]. In this study, we are going to find out some alternative drugs for the treatment of APA according to its gene expression pattern. Hence, investigating differences between the expression patterns of patients with APA and those with NFAA may improve our understanding of the pathogenesis of this disease and provide new clues for the treatment of APA. The workflow of the study is shown in Figure 1.

## 2. Materials and Methods

### 2.1. Microarray Data Information and Data Preprocessing

Raw microarray data (GSE33371, GSE28476, and GSE10927) of the Affymetrix platform (GPL570) were downloaded from the GEO database [11–13]. Twenty-eight adrenocortical adenoma samples from GSE33371 and GSE28476 were integrated as a combined dataset for further analysis, including 10 samples of APA, 12 samples of cortisol-producing adrenocortical adenoma (CPA), and 6 samples of NFAA. Thirteen adrenocortical adenoma samples from GSE10927 were used as a validation dataset. All analyses were performed on R software (version 4.0.0). Probe sets were annotated using the getGEO function of the GEOquery package. Prior to differential gene expression analysis and weighted gene coexpression network analysis (WGCNA), probes matching more than one gene were removed, and multiple probes matching one gene were then used for calculating the average expression value.

### 2.2. Identification of DEGs

Both the combined and validation datasets underwent differential expression gene (DEG) analysis. The Robust Multiarray Average algorithm was used to normalize the raw data [14]. The ComBat function of the sva package was applied to eliminate batch effects between GSE33371 and GSE28476 [15]. Principal component analysis (PCA) was used to display the gene expression pattern among CPA, APA, and NFAA of the combined dataset. DEGs analysis were performed on APA vs. NFAA using the limma package with the empirical Bayes method, and those genes with adjusted *P* value <0.05 and |log2(fold change)| ≥ 1 were defined as statistically significant DEGs. *P* values were adjusted using the Benjamini–Hochberg false discovery rate (FDR).

### 2.3. WGCNA Network Construction

The combined dataset was used for construction of a WGCNA network. The coexpression network was constructed using “WGCNA,” an R package for weighted correlation network analysis [16]. The soft thresholding power was calculated using the pickSoftThreshold function of the WGCNA package. To produce a scale-free network, we used a *β* value = 6. A one-step network construction method was used to identify coexpression modules using the blockwiseModules function. The plotDendroAndColors function of the WGCNA package was used to visualize the coexpression modules.

### 2.4. Construction of Module-Clinical Trait Relationships

**Figure 2:**
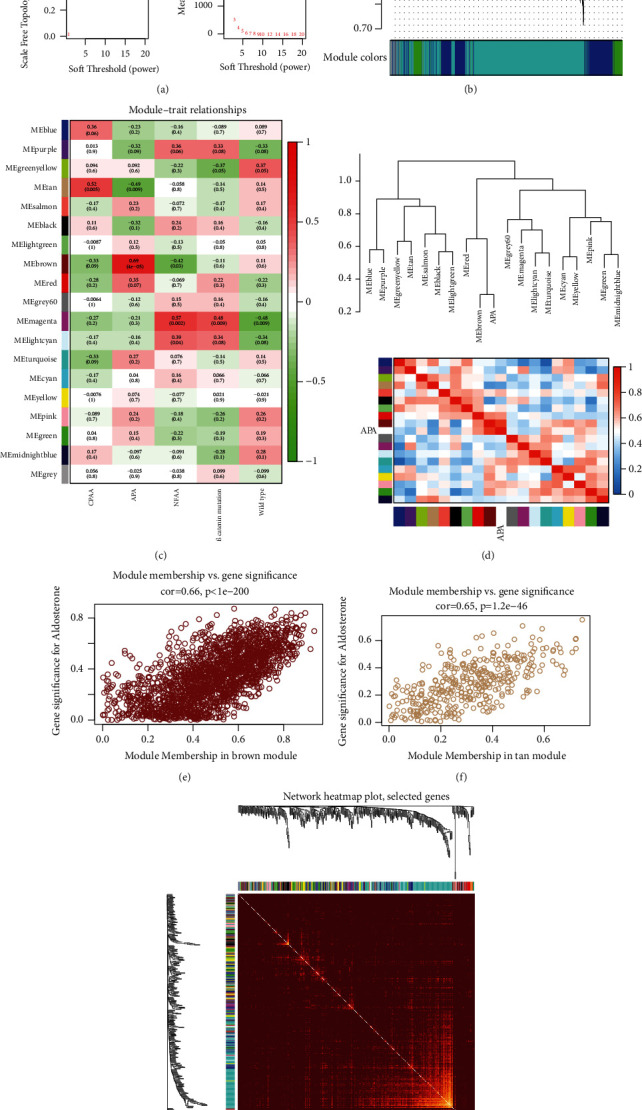
WGCNA analysis of combined datasets. (a) Scale independence and mean network connectivity for soft thresholding powers (*β*). *β* = 6 was selected to achieve model fit maximization. (b) In total, 22,848 genes were assigned to 19 modules, including one gray module, with cutoff of powers = 6. The top image shows a gene dendrogram, and the bottom image shows the gene modules with different colors. (c) Correlation between modules and traits. The upper number in each cell refers to the correlation coefficient of the corresponding module with clinical trait, and the lower number is the corresponding *P* value. (d) The eigengene dendrogram and heat map. The 19 modules are relatively independent, and the brown module was strongly correlated with APA. (e), (f) Scatter plots of GS versus MM for APA in the brown and tan modules. (g) Topological overlap matrix heatmap of 400 genes selected from a total of 22,848 genes. Light color represents high overlap of the genes, and progressively darker color indicates lower overlap. Blocks of light colors along the diagonal represent the coexpression modules.

After the coexpression modules were identified, the module eigengene (ME) was calculated using the first principal component of gene expression level in the corresponding module. Module-clinical trait relationships were assessed using the correlations between MEs and interesting clinical traits including APA, CPA, NFAA, adrenocortical adenoma with *β* catenin mutation, and wild-type adrenocortical adenoma. Module membership (MM) and gene significance (GS) were the two important indices used for determining whether a gene was crucial in each module. Genes with |MM| ≥ 0.8 and |GS| ≥ 0.2 were defined as hub genes of the corresponding module. The brown module contained hub genes with the highest correlation coefficient with APA, while the tan module contained hub genes with the lowest correlation coefficient with APA (Figure 2(c)).

### 2.5. Identification and Functional Enrichment Analysis of Real Hub Genes

Real hub genes were defined as the common genes among DEGs of the combined dataset, hub genes of the brown module and hub genes of the tan module. Real hub gene integration networks were constructed using Cytoscape (version 3.8.0) [17], based on their interrelationship in the WGCNA network. Functional enrichment analysis on the real hub genes was conducted using the clusterProfiler package and Metascape database [18]. Enrichment items with adjusted *P* values <0.05 were considered statistically significant.

### 2.6. Gene Set Enrichment Analysis (GSEA)

We used GSEA, a knowledge-based approach for interpreting genome-wide expression profiles, to analyze the gene expression pattern of the combined datasets [19]. Two groups were examined: APA and NFAA. Only Gene Ontology (GO) or Kyoto Encyclopedia of Genes and Genomes (KEGG) annotations with a nominal *P* < 0.05 were used for further analysis.

### 2.7. Validation Using Other Datasets

Thirteen adrenocortical adenoma samples from GSE10927 were used as the validation dataset. Of these samples, six exhibited hyperaldosteronism and the remaining seven behaved as adrenal masses, without hyperaldosteronism or Cushing syndrome. The expression patterns of the real hub genes in the validation dataset were also identified using DEG analysis. When the DEGs analysis of the validation was performed, the samples exhibiting hyperaldosteronism were defined as the APA group and the samples behaving as adrenal masses were defined as the NFAA group. The expression patterns of the real hub genes in the validation dataset were compared with those of the combined datasets to identify genes with the same expression pattern in both datasets. The results were presented visually using the GraphPad Prism 8 software.

### 2.8. Identification of Potential Candidate Drugs for the Treatment of APA

Putative small-molecule drugs were predicted using the Connectivity Map (CMap) version build 02 (https://portals.broadinstitute.org/cmap/), a database that documents gene expression profiles from cultured human cells treated with bioactive small molecules, which can be used to find out connections between diseases with a particular gene expression pattern and small-molecule drugs that can counteract the diseases in the gene expression level [20]. The DEGs of the combine datasets were used to query the CMap database. The enrichment scores represent the similarity (ranging from −1 to 1) of the gene expression pattern between APA and the cultured human cells treated with the corresponding drugs, a drug inducing similar gene expression pattern with APA will be given a positive score, while a drug producing an opposite pattern will be given a negative score. Hence, a drug with a negative enrichment score was considered to be a putative therapeutic drug for APA.

## 3. Results

### 3.1. Identification of DEGs in the Combined Datasets

**Figure 3:**
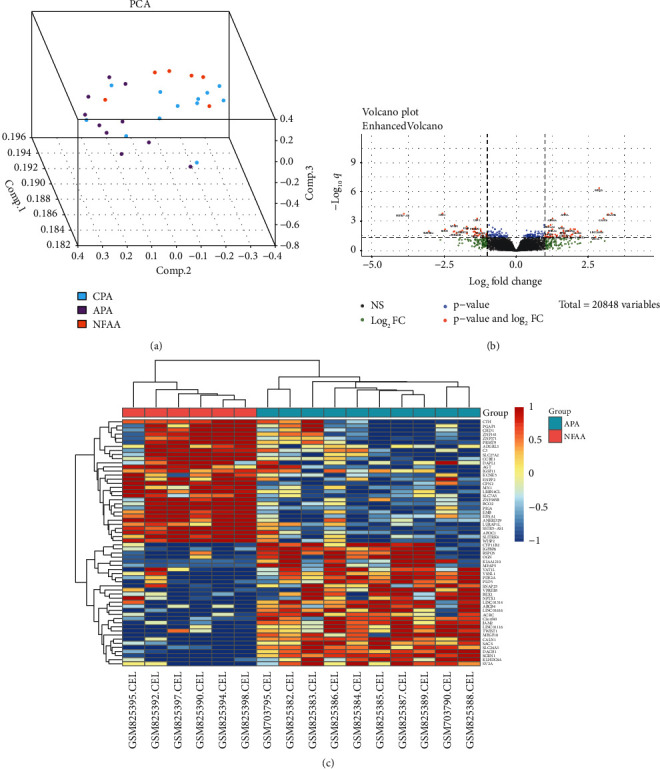
Screening of differentially expressed genes (DEGs) in combined datasets. (a) Spatial graph for PCA of the combined dataset. The light blue spots represent CPA, the purple spots represent APA, and the yellow spots represent NFAA. (b) Volcano map of differently expressed genes between APA tissues and NFAA tissues. (c) Heat map of the top 100 DEGs between the APA and NFAA.

After data preprocessing, PCA was used to analyze the gene expression patterns of the combined datasets. The gene expression pattern of APA was obviously different from that of NFAA, as the spots of APA were spatially far away from NFAA (Figure 3(a)). Eighty-nine DEGs were identified using a cutoff criterion: adjusted *P* value <0.05 and |log2(fold change)| ≥ 1. These DEGs included 52 genes upregulated and 47 genes downregulated in the APA as compared with NFAA. A volcano plot shows all the differently expressed genes between APA and NFAA (Figure 3(b)). The heat map including the 50 most upregulated and downregulated genes clearly shows the difference between APA and NFAA (Figure 3(c)).

### 3.2. Coexpression Analysis

After normalization and batch effect elimination, the combined dataset was used for WGCNA. The scale independence and mean network connectivity for soft thresholding powers (*β*) are shown in Figure 2(a). *β* = 6 was selected to maximize the model fit, and 19 modules were identified, including one gray module (Figure 2(b)). Of all those modules, the brown module exhibited the highest positive correlation with APA (module-trait weighted correlation = 0.69), whereas the tan module showed the strongest negative correlation with the APA (module-trait weighted correlation = −0.49). We also observed that the tan module correlated most strongly with CPA, whereas the magenta module correlated most strongly with adrenocortical adenoma with mutations of the *β*-catenin gene, which occur frequently in adrenocortical adenoma and carcinomas.

An eigengene dendrogram and heat map were used to quantify module similarity and correlation with the clinical trait of APA (Figure 2(d)). The results suggested that there was a high level of independence among the modules. Both the brown and tan modules were selected, and the relationship between GS and MM for APA in both modules is shown on the scatterplot (Figures 2(e) and 2(f)). Genes in both modules highly correlated with APA. For the brown module, *P* < 1*e* − 200 and correlation = 0.66; for the tan module, *P* < 1.2*e* − 46 and correlation = 0.65. Seventy genes from the brown module and 12 genes from the tan module were extracted as hub genes for further analysis, using the criteria MM ≥ 0.8 and GS ≥ 0.2. Finally, 400 genes were selected at random from a total of 20,848 genes to construct a network heatmap plot (Figure 2(g)).

### 3.3. Identification and Functional Enrichment Analysis of Real Hub Genes

**Figure 4:**
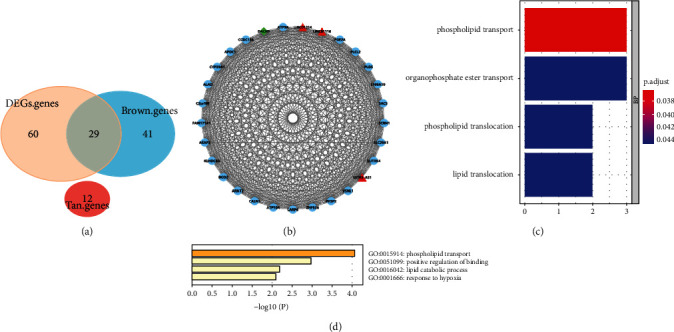
Identification of real hub genes and functional enrichment. (a) The Venn diagram of DEGs of the combined dataset and hub genes from the brown and tan modules. Twenty-nine real hub genes were identified. (b) The interaction network of lncRNA, transcription factor, and coding mRNA for the real hub genes. The red triangles represent lncRNA, the green diamond represents the transcription factor, and the other light blue circles represent the protein coding genes. (c), (d) The results of functional enrichment analysis using the clusterProfiler package and Metascape database. Phospholipid transport was enriched in both methods.

**Figure 5:**
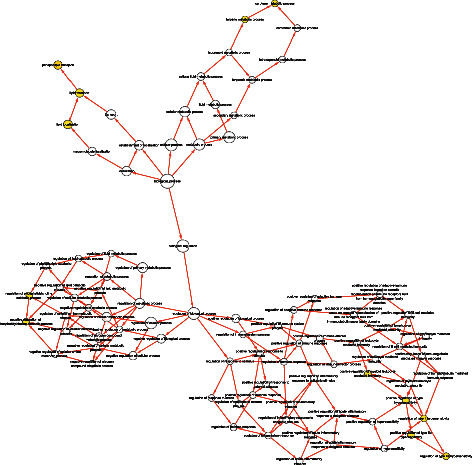
Enrichment network of the real hub genes using the BiNGO plug-in module of Cytoscape; nodes with yellow color indicate significant *P* values for the corresponding GO items; the size of the nodes indicates the number of genes enriched in the corresponding GO items; the bigger the size of the nodes, the more the genes were enriched in the corresponding GO items.

The real hub genes were an intersection of genes set among DEGs of the combined dataset, hub genes in the brown module and hub genes in the tan module. Twenty-nine real hub genes were confirmed (Figure 4(a)), including three lncRNA genes (*SSTR5-AS1, LINC01314*, and *LINC01116*) and one transcription factor (*DACH1*). An integration network of the real hub genes was extracted from the coexpression network using Cytoscape software (Figure 4(b)). To evaluate the function of these genes, GO functional enrichment analyses were performed using the clusterProfiler package and the Metascape database (Figures 4(c) and 4(d)). Four biological processes that met the selection criteria (Section 2) were filtered out, GO:0015914 (phospholipid transport), GO:0015748 (organophosphate ester transport), GO:0045332 (phospholipid translocation), and GO:0034204 (lipid translocation), using the clusterProfiler package; similarly, the enrichment results from the Metascape database were as follows: GO:0015914 (phospholipid transport), GO:0051099 (positive regulation of binding), GO:0016042 (lipid catabolic process), and GO:0001666 (response to hypoxia). We constructed a GO enrichment network on Cytoscape using the BiNGO plug-in module in which nodes with yellow color were significant enrichment GO items (Figure 5).

### 3.4. GSEA Results

**Figure 6:**
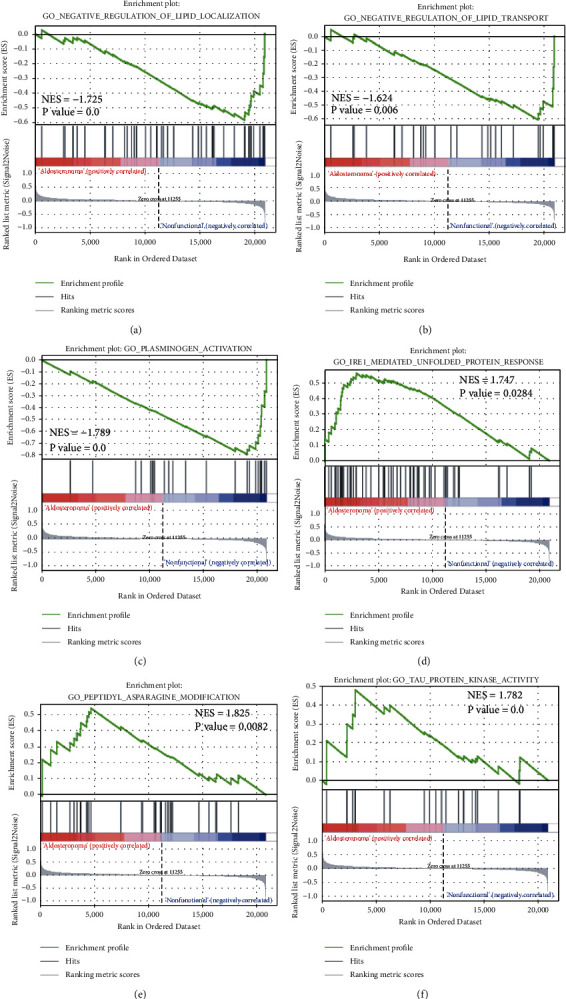
GSEA analysis for GO enrichment using combined datasets. IRE1, an endoplasmic reticulum stress sensor.

**Figure 7:**
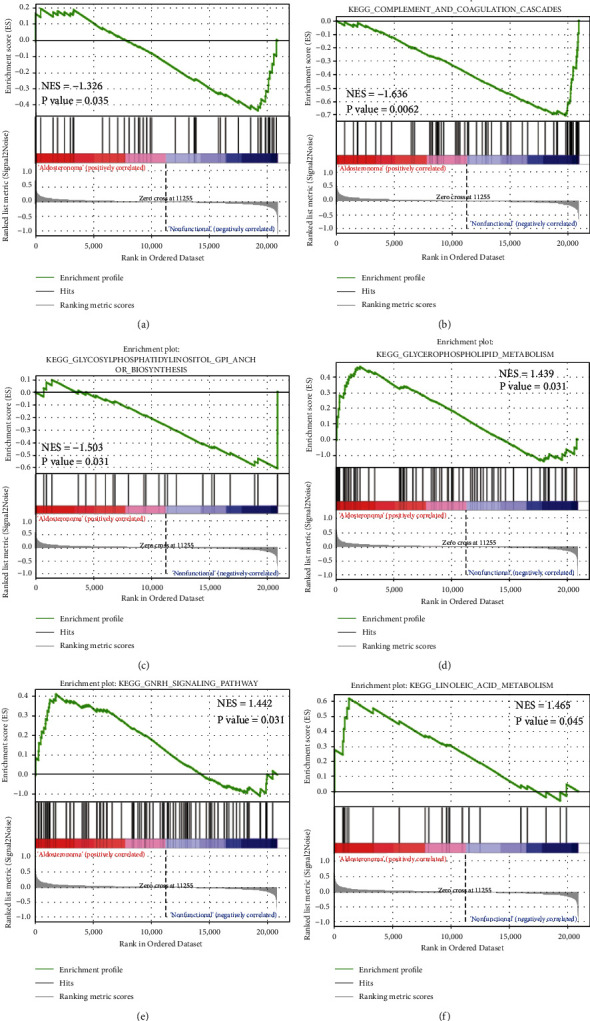
GSEA analysis for KEGG pathway enrichment using combined datasets. ABC, ATP-binding cassette; GPI, glycosylphosphatidylinositol; and GNRH, gonadotropin-releasing hormone.

We performed GSEA for the combined datasets. For GO enrichment analysis, 39 gene sets were enriched in the APA phenotype with *P* values <0.01, whereas 58 gene sets were enriched in the NFAA phenotype with *P* values <0.01. As for KEGG pathway enrichment analysis, four gene sets were enriched in the APA phenotype with *P* value <0.05, whereas seven gene sets were enriched in the NFAA phenotype with *P* value <0.05. Partial results are shown in Figures 6 and 7. GO enrichment analysis revealed that the datasets “negative regulation of lipid localization,” “negative regulation of lipid transport,” and “plasminogen activation” were enriched in the NFAA phenotype, whereas the datasets “IRE-mediated unfolded protein response,” “peptidyl asparagine modification,” and “TAU protein kinase activity” were enriched in the APA phenotype. In the KEGG pathway enrichment analysis, the datasets “ABC transporters,” “complement and coagulation cascades,” and “glycosylphosphatidylinositol GPI anchor biosynthesis” were enriched in the NFAA phenotype, whereas the datasets “glycerophospholipid metabolism,” “GnRH signaling pathway,” and “linoleic acid metabolism” were enriched in the APA phenotype.

### 3.5. Validation of Real Hub Genes Using GSE10927

**Figure 8:**
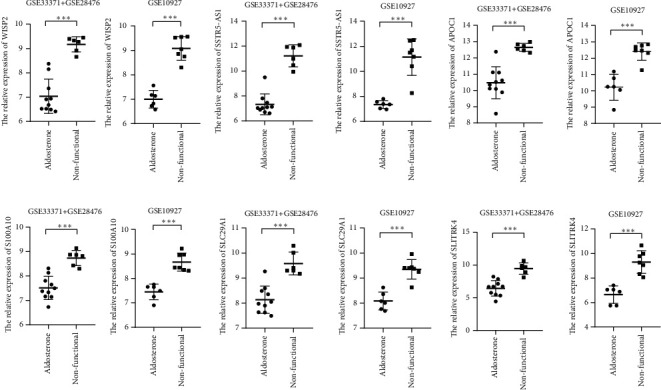
Validation of the expression pattern of real hub genes between the combined datasets (GSE333714 and GSE28476) and the validation dataset (GSE10927). Six genes, including one lncRNA gene, were found to have the same expression pattern between the combined and validation datasets. In addition, all of these genes were poorly expressed in the APA samples compared to the NFAA samples. The three asterisks represent *P* values <0.001 between the two groups.

We used another dataset GSE10927 to validate the real hub genes. Seventeen DEGs of the validation dataset that met the setting criteria (Section 2) were filtered out. The gene expression pattern of these DEGs was compared with that of the previously identified 29 real hub genes of the combined dataset. Six genes—*WISP2, S100A10, SSTR5-AS1, SLC29A1, APOC1*, and *SLITRK4*—with the same expression pattern in the two datasets were identified (Figure 8). All these genes showed relatively low expression in APA samples and high expression in NFAA samples, with all *P* values <0.001.

### 3.6. Screening of Candidate Small-Molecule Drugs for the Treatment of APA

**Figure 9:**
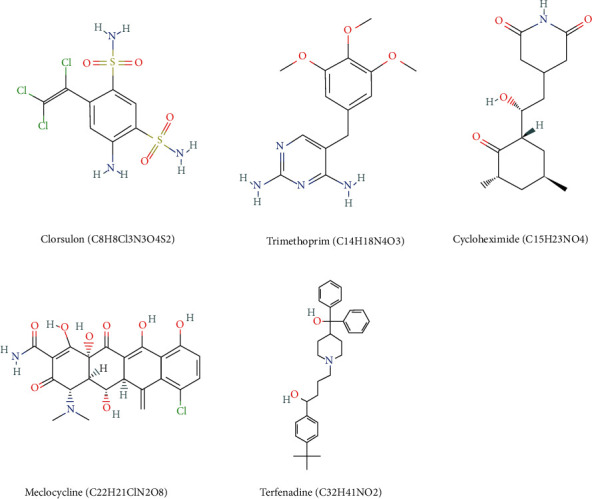
2D structure of putative candidate small-molecule drugs against APA.

Under the screening conditions of enrichment scores <−0.75 and *P* value <0.01, five candidate drugs with potential therapeutic value for APA were filtered out (Table 1), including clorsulon (C8H8Cl3N3O4S2), trimethoprim (C14H18N4O3), cycloheximide (C15H23NO4), meclocycline (C22H21ClN2O8), and terfenadine (C32H41NO2). All of these drugs showed significantly negative correlations with the gene expression pattern in APA, which indicated that these pharmaceuticals may be effective drugs against APA. We also investigated the 2D structure of these candidate drugs using the Pubchem database (https://pubchem.ncbi.nlm.nih.gov/), as shown in Figure 9.

## 4. Discussion

In this study, we combined adrenocortical adenoma samples from two series of GEO datasets to perform differential gene expression analysis and coexpression network analysis. A cluster of real hub genes was identified including three lncRNAs (*SSTR5-AS1, LINC01314*, and *LINC01116*) and one transcriptional regulator (*DACH1*). These genes may play an important role in regulating the expression of other real hub genes. *LINC01116* has been implicated in osteosarcoma, gastric cancer, glioma, and head and neck squamous cell carcinoma, for promoting the proliferation, invasion, and migration of tumor cells [21–23]. We observed that *LINC01116* was highly expressed in NFAA samples and lowly expressed in APA samples, suggesting that it is a protective factor for APA. *DACH1* as a transcription factor is essential for podocyte function [24]. However, the role of *DACH1* in the pathogenesis of APA requires further investigation.

We performed functional enrichment through various methods to further analyze the real hub genes. Using the clusterProfiler package, we identified four highly correlated biological processes: phospholipid transport, organophosphate ester transport, phospholipid translocation, and lipid translocation, of which phospholipid transport is the one with the lowest adjusted *P* value. Phospholipid transport was also found to be a significant enriched item, when using the Metascape database for functional enrichment analysis. After that, GO annotation enrichment was performed on Cytoscape using the BiNGO plug-in module and a GO enrichment network was also constructed. Most significant items in the GO enrichment were related to phospholipid transportation and (or) phospholipid metabolic processes. Taken together, these results suggest that phospholipid transportation and metabolism may be the pathogenesis of APA.

Similar conclusions were obtained from the results of GSEA enrichment analysis. The enrichment results of GO annotations for the NFAA phenotype included “negative regulation of lipid localization” and “negative regulation of lipid transport,” whereas the enrichment results of the KEGG pathway for the APA phenotype contained glycerophospholipid metabolism, indicating that a high level of phospholipid metabolic activity in APA may differentiate it from NFAA. The underlying molecular mechanisms require further investigation, although the GSEA enrichment results have provided some clues.

Six genes with the same expression pattern in the combined and validation datasets were identified. However, the validation samples had its limitations. Owing to the absence of data about secretory hormones in the validation samples, sample grouping may yield false negative results when dividing the adrenal mass samples to the NFAA group. The hyperaldosteronism samples were certainly APA; however, we cannot ensure that all the adrenal mass samples were NFAA. Although this phenomenon might generate false negative results during sample grouping process, it increased the specificity of the validation results, as only genes with a significant expression difference between APA and NFAA can be defined as DEG using the above screening criteria (Section 2).

Six genes—*WISP2, S100A10, SSTR5-AS1, SLC29A1, APOC1*, and *SLITRK4*—were identified having the same expression pattern in the combined and validation datasets. These genes play various roles in biological processes in which WISP2, S100A10, and APOC1 indirectly or directly participate in lipid metabolism. WISP2 is a novel adipokine, and it has been reported that WISP2 knockdown enhanced adipogenesis [25]. S100A10 is a cell membrane repair protein holding a complex interaction relationship with the phospholipid membrane [26]. *SSTR5-AS1*, an antisense lncRNA of *SSTR5L*, acts as a tumor suppressor, as well as a potential biomarker for antitumor therapy [27]. The human equilibrative nucleoside transporter 1 (*hENT1*), also called *SLC29A1*, a member of the SLC29 family, plays crucial roles in adenosine signaling, nucleoside-derived anticancer, and antiviral drug transport in humans [28]. Apolipoprotein C1 (*APOC1*), the smallest one in all of the apolipoproteins, participates in lipid transport and metabolism. It associates with triglyceride-rich lipoproteins and high-density lipoproteins and exchanges esterified cholesterol between lipoprotein classes, thereby controlling the plasma levels of lipids. Gautier's research suggests that human *APOC1* transgenesis reduces atherogenesis in hypercholesterolemic rabbits [29]. *APOC1* was also a key gene enriched, using the clusterProfiler package and Cytoscape (Tables 2 and 3), further confirming that phospholipid transportation and phospholipid metabolism may play an important role in the pathogenesis of APA.

Among all the candidate drugs speculated using the CMap database according to the gene expression file of DEGs, cycloheximide was the one that had been reported on suppressing adrenocortical activity [30]. Researchers had found that cycloheximide carried an inhibiting effect on the biosynthesis of total phospholipids [31]. Kempen's study suggested that cycloheximide not only suppresses very-low-density lipoprotein (VLDL) secretion but also inhibits the release of other apolipoproteins [32]. In an earlier study, phospholipid production was found to be severely inhibited in cycloheximide-treated cells [33]. A variety of evidence suggests that cycloheximide plays a role as an inhibitor for phospholipid biosynthesis. According to our study above, this countereffect of cycloheximide to phospholipid biosynthesis may be the pathophysiology basis of its inhibitory effect to APA. Whether the other candidate drugs also hold the properties like cycloheximide still needs more study to confirm. Furthermore, trimethoprim shows an ability to bind to the phospholipid matrix of the bilayer membrane [34], which indicates that trimethoprim may also be a potential effective drug for APA.

## 5. Conclusions

Using differential gene expression analysis and WGCNA, we identified specific real hub genes for APA. Three of these genes encoded lncRNAs, and one of them encoded a transcription factor. We performed functional enrichment analysis using these real hub genes. Most of the enrichment items were related to phospholipid metabolism. Additionally, we found out some promising candidate drugs for the treatment of APA using the CMap database, one of which had been reported on suppressing adrenocortical activity and was also a potent inhibitor for phospholipid biosynthesis. These results suggest that phospholipid metabolism is an important mechanism that may control aldosterone production and (or) secretion from APA. Therefore, drugs with the ability to regulate the phospholipid metabolic process on adrenal tissue may be promising medications for APA. However, the number of samples used in this study was small, a limitation which might bias our conclusions. Hence, more studies with larger sample sizes are required in future.

## Figures and Tables

**Table 1:** The potential small-molecule drugs.

Cmap name	Mean	*n*	Enrichment score	*P* value	Specificity	Percent nonnull
Clorsulon	−0.588	4	−0.843	0.00105	0.0071	100
Trimethoprim	−0.546	5	−0.77	0.00118	0	100
Cycloheximide	−0.494	4	−0.769	0.00571	0.0741	100
Meclocycline	−0.443	4	−0.751	0.00772	0	75
Terfenadine	−0.575	3	−0.839	0.00843	0.0642	100

**Table 2:** GO enrichment analysis of real hub genes using the clusterProfiler package

GO ID	GO terms	*q* value	Gene symbol
0015914	Phospholipid transport	0.0274	*APOC1/ATP10A/ATP9A*
0015748	Organophosphate ester transport	0.034	*APOC1/ATP10A/ATP9A*
0045332	Phospholipid translocation	0.045	*ATP10A/ATP9A*
0034204	Lipid translocation	0.045	*ATP10A/ATP9A*

**Table 3:** GO enrichment analysis of real hub genes using the Cytoscape software.

GO ID	GO terms	*q* value	Gene symbol
15914	Phospholipid transport	0.004	*APOC1/ATP10A/ATP9A*
6869	Lipid transport	0.042	*APOC1/ATP10A/ATP9A*
10876	Lipid localization	0.042	*APOC1/ATP10A/ATP9A*
42214	Terpene metabolic process	0.042	*BCO2*
10899	Regulation of the phosphatidylcholine catabolic process	0.042	*APOC1*
10900	Negative regulation of the phosphatidylcholine catabolic process	0.042	*APOC1*

## Data Availability

The datasets generated and/or analyzed during the current study are available from the Gene Expression Omnibus data repository (https://www.ncbi.nlm.nih.gov/geo/) with the accession numbers GSE33371, GSE28476, and GSE10927.
